# AAV vector distribution in the mouse respiratory tract following four different methods of administration

**DOI:** 10.1186/s12896-017-0365-2

**Published:** 2017-05-15

**Authors:** Lisa A. Santry, Joelle C. Ingrao, Darrick L. Yu, Jondavid G. de Jong, Laura P. van Lieshout, Geoffrey A. Wood, Sarah K. Wootton

**Affiliations:** Department of Pathobiology, University of Guelph, Guelph, Ontario N1G 2W1 Canada

**Keywords:** Adeno-associated virus, Vector instillation, Transgene expression, Intratracheal, Intranasal, Intubation, Respiratory tract, Respiratory epithelium

## Abstract

**Background:**

Targeted delivery of gene therapy vectors to the mouse respiratory tract is often performed via intranasal or intratracheal administration; however, there can be a great deal of variability between these methods, which could potentially influence experimental results. Improving the accuracy and precision of lung delivery will not only reduce the number of animals required to detect statistically significant differences, but may reduce the variability of studies from different laboratories.

**Results:**

Here we evaluated three different methods of adeno-associated virus (AAV) vector administration to the respiratory tract in mice (intranasal, intubation, and intratracheal injection) and discuss the advantages, challenges, and shortcomings of each. We also present a modified-intranasal delivery technique that is superior to passive administration of vector into the nares of anesthetized supine animals. Transgene expression was consistently visible in the nasal cavity, trachea, and proximal to middle aspect of all lung lobes for all four methods, whereas transgene expression was consistently observed in the most distal aspect of lung lobes only with the intubation and intratracheal injection techniques. AAV vector genome copy numbers in the lung were approximately four-fold lower in mice that received vector via intranasal administration in comparison to the other three methods of vector delivery. The modified intranasal, intubation and intratracheal injection methods of vector administration did not yield statistical differences in AAV vector genome copy numbers in the lung. With regard to reproducibility of vector distribution within and between animals, the modified-intranasal technique was superior.

**Conclusion:**

Our results show that mode of AAV vector administration to the murine respiratory tract should be selected based on desired target site and skill of the researcher, and that appropriate technique selection may greatly influence experimental outcomes.

**Electronic supplementary material:**

The online version of this article (doi:10.1186/s12896-017-0365-2) contains supplementary material, which is available to authorized users.

## Background

Gene therapy is currently being evaluated for a wide range of acute and chronic lung diseases including those caused by single gene defects such as cystic fibrosis, alpha-1 antitrypsin deficiency and surfactant protein B deficiency [1]. While the lung provides a natural route of entry into the host and can be accessed in a comparatively easy and noninvasive manner, methods for consistent and efficient delivery of viral vectors to the respiratory tract are required. There have been a number of studies evaluating variables such as volume of inoculum, effect of diluent, nature of anesthesia (inhaled vs. injected), and position of mouse [2, 3] and how these affect intranasal delivery of therapeutic substances in mouse models, but a comparison of different delivery methods using viral vectors has not been done. Targeted delivery of viral vectors to the mouse respiratory tract can be performed by a variety of different methods, some of which require extensive skill and training. In this report, four different methods of delivering viral vectors to the mouse respiratory tract were evaluated for ease and consistency, vector distribution, and invasiveness of the procedure with the aim of identifying the most effective and consistent method of delivery requiring minimal skill development.

**Fig. 1:**
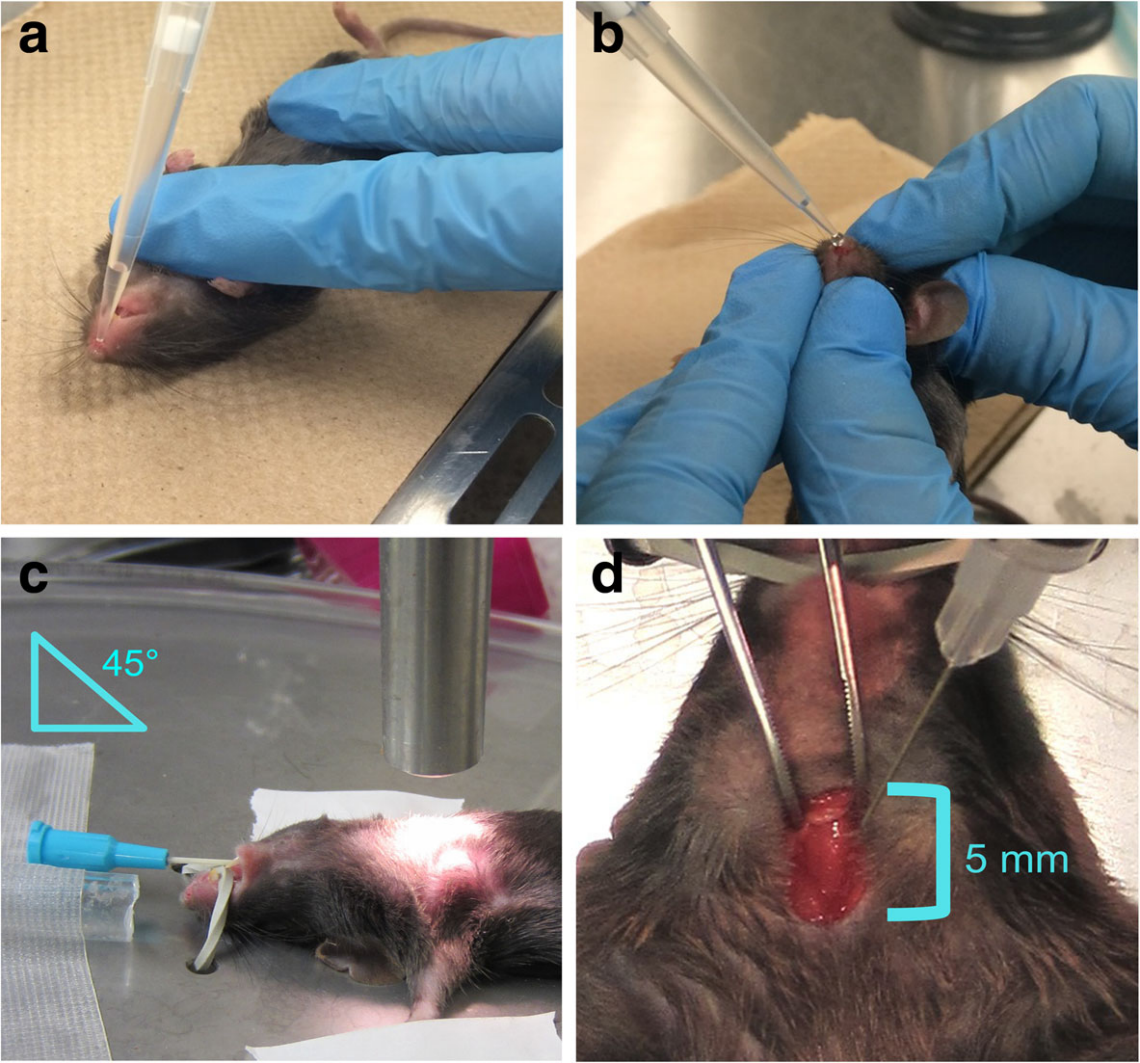
Overview of the four different delivery methods used to administer viral vector to the mouse respiratory tract. **a** Intranasal technique. Mice were anesthetized with isoflurane and the viral vector administered drop wise over the nares allowing for passive inhalation of the substance. **b** Modified-intranasal technique. Mice were anesthetized with isoflurane and the viral vector administered drop wise over the nares. The mouth is covered during administration to promote inhalation through the nose and subsequent distribution of the vector throughout the lower respiratory tract as well as to prevent swallowing of the administered substance. **c** Intubation technique. Mice were anesthetized with isoflurane in oxygen and a 22-gauge catheter placed into the trachea. The viral vector was administered to the airways through the catheter. **d** Intratracheal injection technique. Mice were anesthetized with isoflurane and the viral vector injected directly into the trachea following surgical visualization

For this study, a lung-tropic AAV serotype 6 vector [4] expressing the human placental alkaline phosphatase (hPLAP) reporter gene was administered to the respiratory tract of C57BL/6 mice by intranasal, modified intranasal, endotracheal intubation, or intratracheal injection (Fig. 1) and subsequently evaluated for transduction efficiency. A total of 1x10^11^ vector genomes (vg) were delivered in a fixed volume of 80 μl (delivered either in 2 x 40 μl doses as in the case of intranasal and modified intranasal administration or one dose of 80 μl as in the case of intubation and intratracheal administration) using isoflurane as the anesthetic.

Here we show that the modified-intranasal technique resulted in a similar and consistent distribution of vector to the upper and lower respiratory tract, including all lung lobes, in all mice examined. While the intratracheal and intubation methods resulted in somewhat inconsistent distribution of vector, particularly to one lung lobe, the efficiency of lung gene delivery was similar to that of the modified intranasal technique. However, only the intubation method resulted in consistent vector transduction of the distal most aspect of the lung. Intranasal delivery efficiently targeted vector to the upper respiratory tract and as expected, led to robust transduction of the nasal epithelium with significantly less vector transduction in the lung relative to the other three methods of vector administration. In conclusion, the modified-intranasal technique proved to be the most efficient and consistent method of vector delivery requiring the least amount of skill. Use of the modified-intranasal technique for delivery of viral vectors to the mouse respiratory tract may improve the accuracy and precision of lung delivery, which may reduce variability between studies from different laboratories and can have animal welfare implications by reducing the number of animals required for detecting statistically significant differences.

## Results

### Gross analysis of transgene expression

**Fig. 2:**
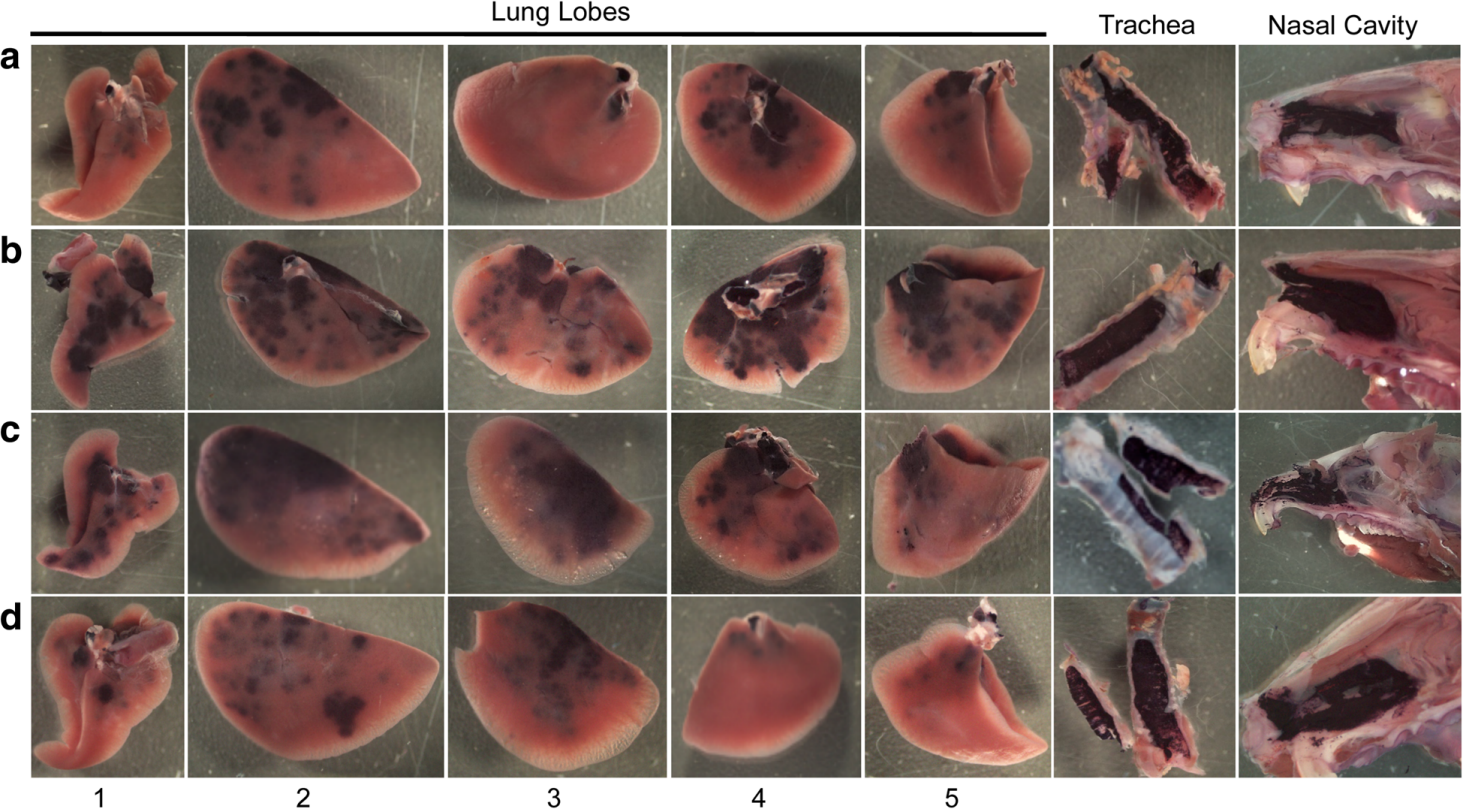
Gross images of alkaline phosphatase staining in the upper and lower respiratory tract of mice following intranasal administration of an AAV vector expressing hPLAP. AAV vector (1x10^11^ vg) was administered by the intranasal route to 7-week-old C57BL/6 mice and tissues evaluated for hPLAP expression after three weeks. The intranasal technique resulted in intense hPLAP staining of the upper respiratory tract, including the nasal cavity and trachea, and only the most proximal aspect of the lower respiratory tract, with similar transduction efficiencies observed for all mice in the group. Letters **a-d** represent individual mice and numbers one through five indicate the accessory lobe (1), the left lobe (2), the right cranial lobe (3), the right caudal lobe (4) and the right middle lobe (5)

**Fig. 3:**
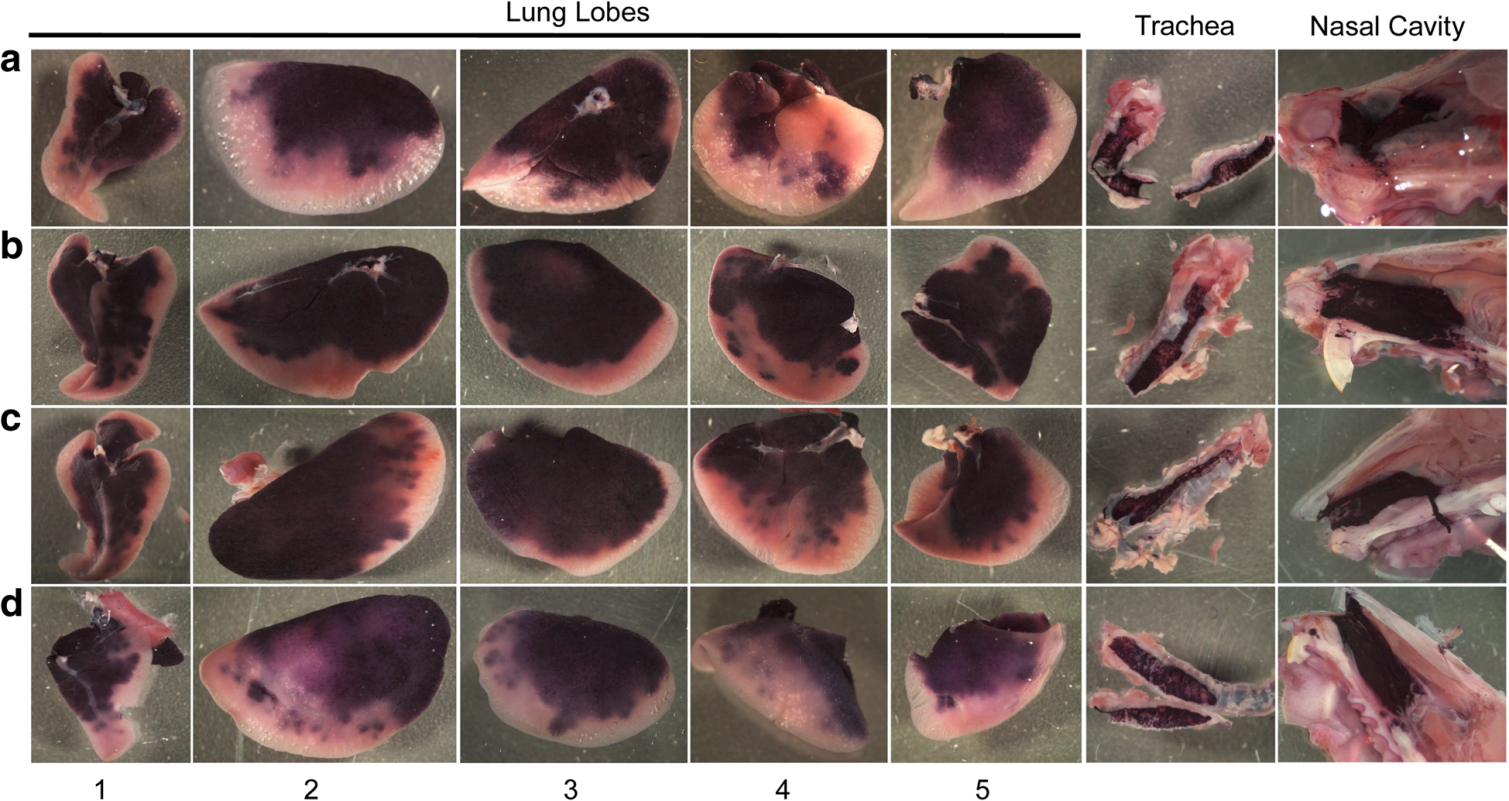
Gross images of alkaline phosphatase staining in the upper and lower respiratory tract of mice following administration of an AAV vector expressing hPLAP using the modified intranasal technique. AAV vector (1x10^11^ vg) was administered by the modified intranasal method to 7-week-old C57BL/6 mice and tissues evaluated for hPLAP expression after three weeks. The modified intranasal technique resulted in intense staining of the upper respiratory tract, including nasal epithelium and trachea, as well as the proximal and mid-distal aspect of the lower respiratory tract in all mice examined. Letters **a-d** represent individual mice and numbers one through five indicate the accessory lobe (1), the left lobe (2), the right cranial lobe (3), the right caudal lobe (4) and the right middle lobe (5)

**Fig. 4:**
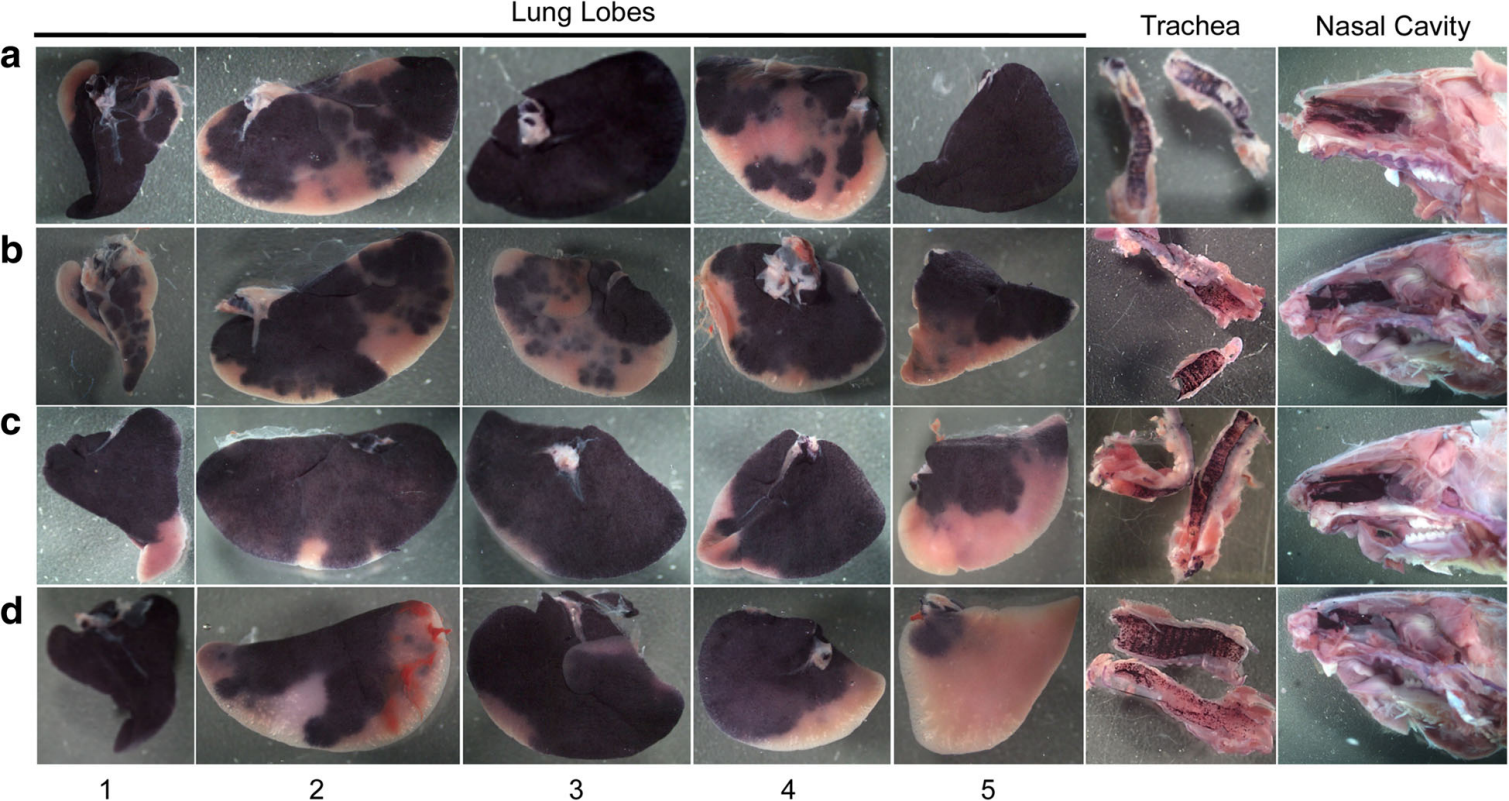
Gross images of alkaline phosphatase staining in the upper and lower respiratory tract of mice following administration of an AAV vector expressing hPLAP using the intubation technique. AAV vector (1x10^11^ vg) was administered by endotracheal intubation to 7-week-old C57BL/6 mice and tissues evaluated for hPLAP expression after three weeks. Injection via intubation resulted in somewhat variable hPLAP staining of the upper and lower respiratory tract, including the distal aspect of the lung. Staining in the nasal epithelium and trachea was not as intense as with the intranasal methods. Note that some lung lobes were highly transduced with vector distribution throughout the entire lung lobe (see A3, A5 and D1). Letters **a-d** represent individual mice and numbers one through five indicate the accessory lobe (1), the left lobe (2), the right cranial lobe (3), the right caudal lobe (4) and the right middle lobe (5)

**Fig. 5:**
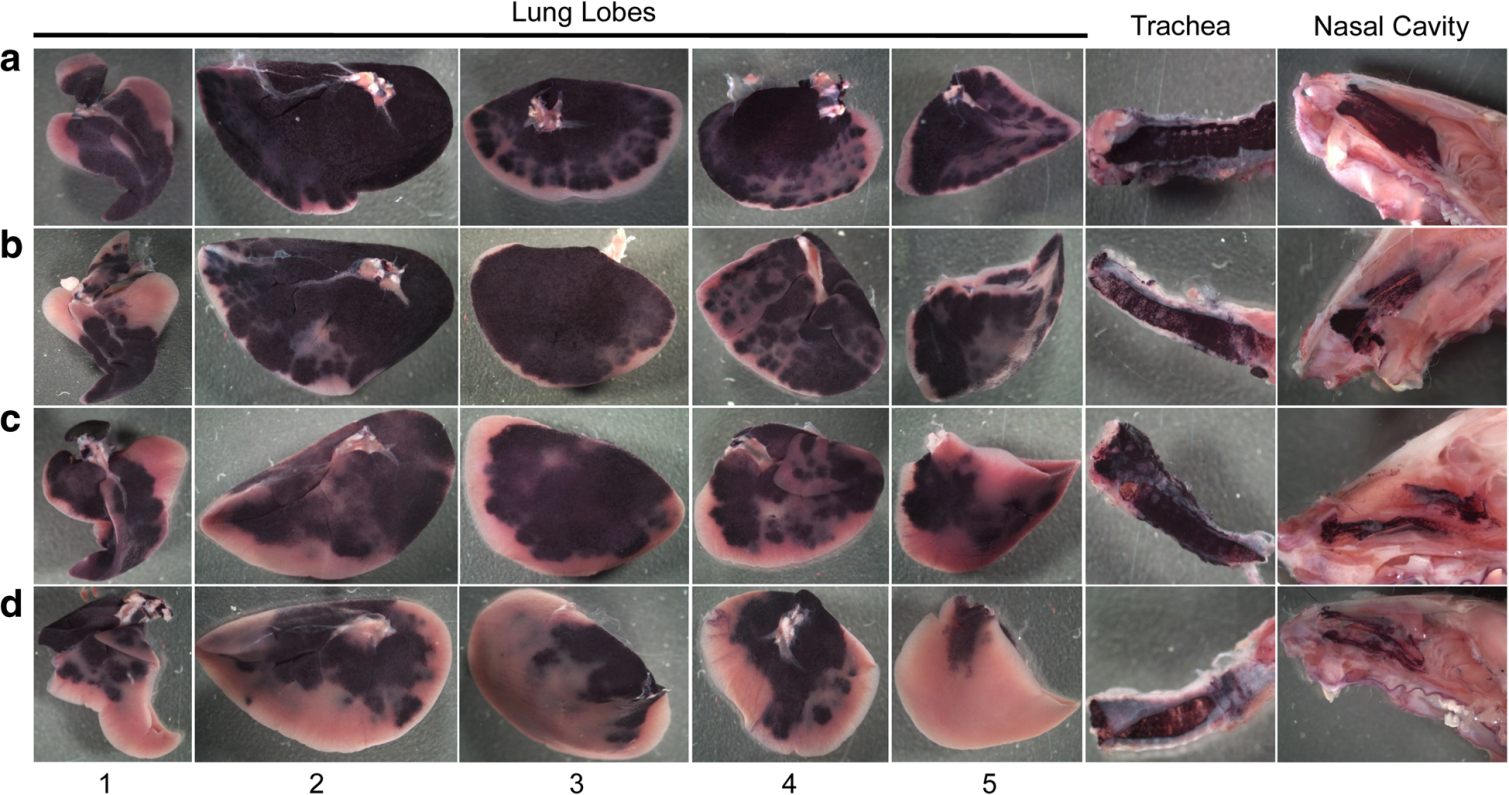
Gross images of alkaline phosphatase staining in the upper and lower respiratory tract of mice following intratracheal administration of an AAV vector expressing hPLAP. AAV vector (1x10^11^ vg) was administered by intratracheal injection to 7-week-old C57BL/6 mice and tissues evaluated for hPLAP expression after three weeks. Intratracheal injection resulted in intense hPLAP staining in the trachea with some staining in the nasal epithelium. This delivery method resulted in variable hPLAP expression in the lower respiratory tract, such that the proximal and mid-distal aspect of the lower respiratory tract were well transduced in three (**a**, **b** and **c**) of four mice and in two of the four mice, hPLAP staining was observed in the distal aspect of some of the lung lobes, mainly the largest lung lobe (**a** and **b**). Letters **a-d** represent individual mice and numbers one through five indicate the accessory lobe (1), the left lobe (2), the right cranial lobe (3), the right caudal lobe (4) and the right middle lobe (5)

Grossly, hPLAP transgene expression was visible in all nasal cavities, trachea, and the proximal to middle aspect of all lung lobes (Figs. 2, 3, 4, 5) for all four methods of vector administration. The intranasal technique resulted in staining of the proximal aspect of the lower respiratory tract with little to no staining of mid to distal aspects of lung lobes, respectively (Fig. 2). The modified-intranasal technique resulted in hPLAP staining in the proximal to mid-distal aspect of the lower respiratory tract, with no staining of the most distal aspects of lung lobes (Fig. 3). The intubation (Fig. 4) and intratracheal injection (Fig. 5) techniques resulted in hPLAP staining of the lower respiratory tract, up to and including distal aspects of lung lobes. For the modified-intranasal technique, hPLAP expression was consistent between locations and between animals, whereas the intubation method resulted in markedly less hPLAP expression observed grossly in the middle lung lobe of half of the animals (Fig. 4c and d, lung lobe #5). In one of four animals subjected to intratracheal administration of vector, hPLAP staining was restricted to the proximal aspect of the lower respiratory tract (Fig. 5d, lung lobe #5). While more robust hPLAP expression was observed grossly in the nasal cavity following intranasal (Fig. 2) and modified intranasal delivery of vector (Fig. 3), both the intubation and intratracheal delivery methods resulted in varying degrees of hPLAP expression in the nasal cavity (Figs. 4-5).

### Histological analysis of AAV vector distribution in the mouse lung

**Fig. 6:**
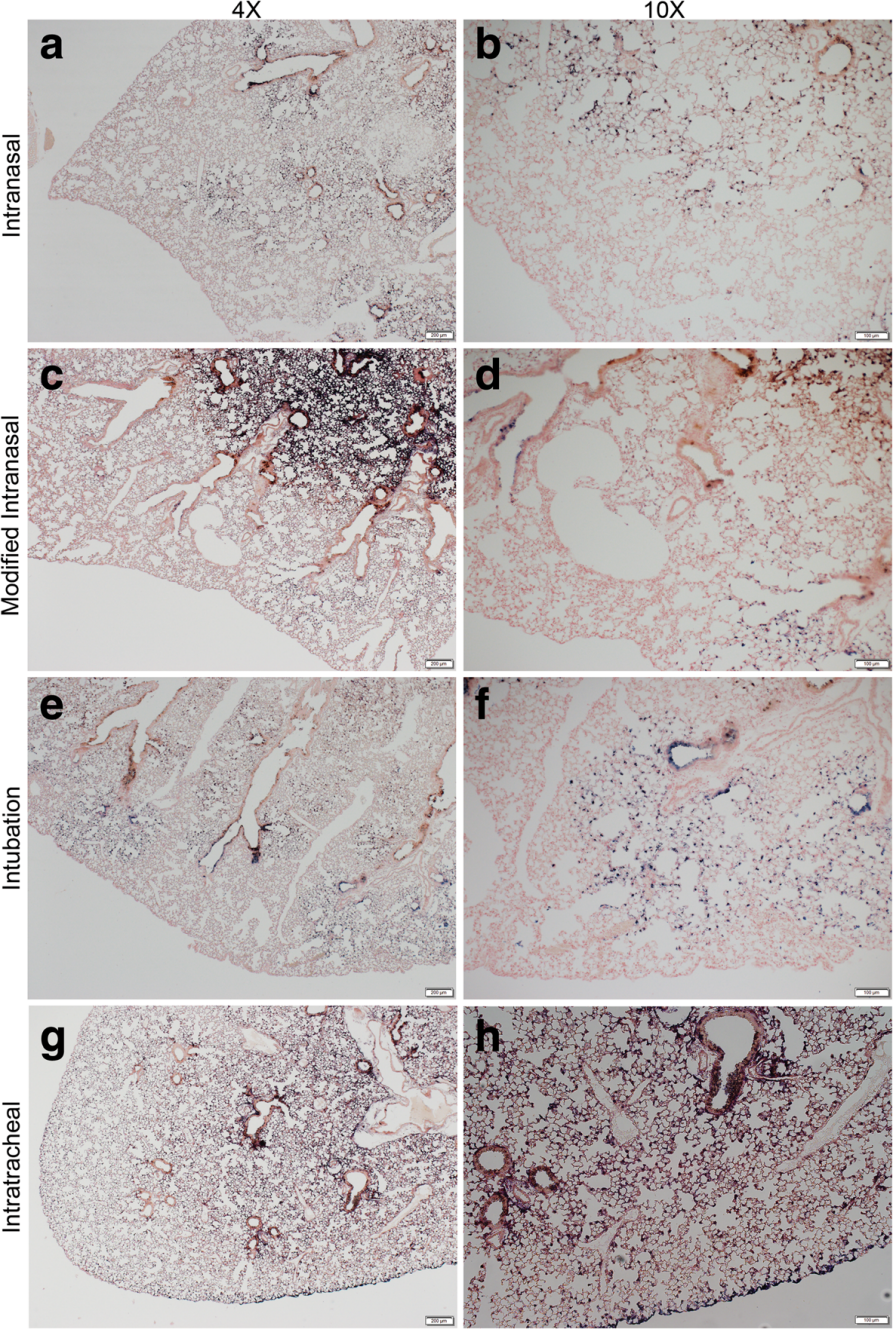
Histological analysis of alkaline phosphatase staining in the lungs of mice 21 days after delivering 1x10^11^ vg of an AAV6 vector expressing hPLAP using four different methods of administration. Viral vectors were administered by the intranasal (**a** and **b**), modified-intranasal (**c** and **d**), intubation (**e** and **f**), or intratracheal injection (**g** and **h**) method. Tissue sections of one representative lung lobe stained for hPLAP expression and counterstained with nuclear fast red are shown at 4x (A, C, E, F; scale bar = 200 μM) and 10x (B, D, F, H; scale bar = 100 μM) magnification

Efficiency of vector distribution in the lung at 21 days post-delivery of 1x10^11^ vg of an AAV6 vector expressing hPLAP by the intranasal, modified intranasal, intratracheal and intubation methods was evaluated histologically (Fig. 6 and Additional file 1 Figure S1, Additional file 2 Figure S2, Additional file 3 Figure S3, Additional file 4 Figure S4, Additional file 5 Figure S5,). Representative sections of paraffin-embedded lung tissue subjected to staining for hPLAP expression are shown in Figs. 6a-h. Histologically, AP staining was observed in bronchiole, bronchiolar, and alveolar epithelial cells in all mice for all techniques (Fig. 6a-h). Consistent with gross observations, a considerable amount of AP staining was observed in distal alveolar epithelial cells with the intubation (Fig. 6e and f) and intratracheal injection (Fig. 6g and h) techniques. In addition, AP expression was predominantly detected within the proximal airway following the modified intranasal technique. For all methods, AP expression was consistent between locations and between animals.

### Quantification of vector genome copy number in lungs

**Fig. 7:**
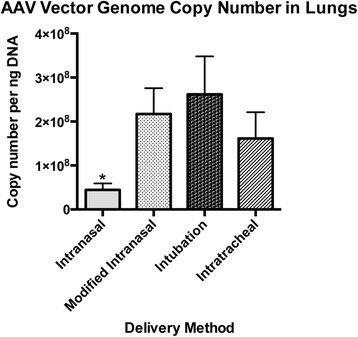
Vector genome copy numbers in lung tissue. AAV genome copy numbers/ug of genomic DNA were determined 21 days after administration of 1x10^11^ vg of an AAV6 vector expressing hPLAP. Genomic DNA was isolated from lung tissue slices tissues and 100 ng of each was used in triplicate to determine vector genome copies using Taqman qPCR. Levels of significance were determined using one-way analysis of variance followed by Tukey’s post-hoc test. The data are shown as mean values ± SEM. ^*^ *P* < 0.05

We further evaluated the efficiency of AAV6 vector transduction via the four different delivery methods by quantifying the number of vector genomes in the lungs of each of the experimentally infected mice at 21 days post-transduction. Vector copy number, which corresponds to viral genomes per ng of genomic DNA, was quantified using a well-established Taqman qPCR assay [5]. As shown in Fig. 7, the mean copy number of AAV vectors in the lung after intranasal delivery was approximately four-fold lower in comparison to the other three methods of vector delivery. The other three methods of vector delivery did not yield statistical differences in AAV copy number in the lung.

## Discussion

It has been shown that relative distribution of a substance between the upper, lower respiratory, and gastrointestinal tract in mice following intranasal administration is largely dependent on delivery volume and level of anesthesia [2, 3]. Studies have shown that small volumes, specifically those less than 10 μl, resulted in detection of the administered substance predominantly in the upper respiratory tract, whereas efficient detection in the lower respiratory tract required administration of volumes greater than 50 μl [2, 3]. In addition, intranasal installation was more efficient in anesthetized animals versus awake animals [2], and in animals anesthetized with inhalant anesthetic versus parenteral [3]. Based on these findings, the current study evaluated transduction of the respiratory tract of mice following delivery of 1x10^11^ vg of an AAV serotype 6 vector expressing the human placental alkaline phosphatase (hPLAP) reporter gene in a fixed volume of 80 μl to animals that were anesthetized with isoflurane at deep plane in order to contrast and compare four different methods of administration.

The modified-intranasal technique resulted in a similar and consistent distribution of vector to the upper and lower respiratory tract, including all lung lobes, with vector seldom reaching the most distal aspect of the lung. Intratracheal and intubation methods resulted in less consistent vector transduction with a lack of localization to the right middle lung lobe; however, both methods resulted in vector transduction of the distal most part of the lung. The intranasal technique resulted in vector localization primarily within the upper respiratory tract and significantly less vector transduction in the lung compared to the other three methods. These results indicate that the method of vector administration should be chosen based on preferred target site and expertise of the researcher, and that appropriate technique selection may influence experimental outcomes.

The skills and/or amount of training required to perform each technique varied considerably. The intratracheal injection method required basic surgical skills for closure of the skin incision. The intubation method was inconsistent, likely due to the challenging nature of the technique. Both of these techniques required considerable practice in order to ensure the well-being of the animal following recovery, and efficient/consistent administration of the viral vector to the respiratory tract. In contrast, both intranasal delivery methods required minimal training and efficient and consistent transduction was observed in the respiratory tract following a relatively short time commitment to practicing the modified-intranasal technique. See Table 1 for a summary of the advantages and disadvantages of the four different delivery methods evaluated.

**Table 1:** Summary of the advantages and disadvantages of the four delivery methods evaluated

Technique	Anatomic Target	Advantages	Disadvantages
Intranasal	- Nasal epithelium - Tracheal epithelium - Proximal aspect of lung lobes	- Relatively quick - Little skill/training required - Can be performed by one person - Inexpensive - Best option if wanting to target the upper respiratory tract - Can administer multiple doses	- Frequent loss of vector due to swallowing - Inefficient delivery to the lower respiratory tract
Modified intranasal	- Nasal epithelium - Tracheal epithelium - Proximal to middle aspect of lung lobes	- Relatively quick - Little skill/training required - Inexpensive - Least variable delivery method - Can administer multiple doses	- Requires two people - Does not reach the distal aspect of lung lobes
Intubation	- Nasal epithelium - Tracheal epithelium - Proximal, middle and distal aspect of lung lobes	- Method that most consistently reaches the distal aspect of the lung lobes - Can be performed by one person	- Technically challenging - Requires more equipment than the other methods - Variable - Time consuming - Possibility of losing vector if esophagus is penetrated - Excessive swelling prevents repeated attempts at intubation
Intratracheal	- Nasal epithelium - Tracheal epithelium - Proximal to middle aspect of lung lobes - Possible to target distal aspect of lung lobes	- Primarily targets the lower respiratory tract - Limited delivery to the nasal epithelium - Can be performed by one person, but is easier with two people	- Technically challenging - Requires surgical skills - Variable - Invasive - Requires analgesic - Re-administration not advised if done more than 5 days after initial administration

Previous studies have shown that depth of anesthesia can influence the amount of administered substance lost to the gastrointestinal tract [2, 3]. For both the intranasal and the modified-intranasal technique, the mouse was briefly removed from the source of isoflurane anesthesia prior to vector administration. Therefore, quick recovery and a light plane of anesthesia may have resulted in an increased loss of vector to the gastrointestinal tract. According to our criteria, which were established based on previous experiments, swallowing was documented to have occurred if liquid was found pooling in the mouth of the mouse immediately following vector administration or if bubbles of liquid were released from the mouth during or immediately following vector administration. Unfortunately, because we did not harvest the esophagus and stomach from the animals used in this study, we cannot substantiate these observations. Nevertheless, pooling of liquid in the mouth and production of bubbles was commonly observed with the intranasal delivery method and may have contributed to significantly less vector particles being delivered to the lungs. However, modifying this technique such that the mouth was covered and the animal maintained upright at a 45 °C angle during vector administration resulted in no apparent loss of vector into the gastrointestinal tract.

Repeat-administration using the intratracheal injection technique may be challenging. The intratracheal injection technique requires a skin incision, which will begin to heal immediately following closure. If re-administration is required within a relatively short timeframe, the same incision site could be re-opened since healing would be minimal. Greater than seven days following closure will result in significant fibrosis of the incision site, which may make re-incision challenging. The second incision may need to be made slightly lateral to the first; however, this could impede proper visualization of the trachea. For this reason, if repeat administration is required, an alternate method of administration is recommended.

Additional studies are required to evaluate the extent and character of inflammation resulting from each technique. Control mice received saline in order to evaluate the effects of each technique at the tissue level. No changes consistent with trauma, inflammation or repair were observed (data not shown). However, histopathology at several time points immediately following each technique would need to be evaluated in order to properly characterize whether tissue damage or local inflammation resulted. The intratracheal injection technique likely resulted in the most inflammation locally, at the site of incision; however, evaluation of an acute immune response may be confounded by a local immune response at the site of the incision. When performing the intubation technique, considerable swelling of the tongue and throat may occur if repeated attempts at insertion of the catheter are required. This is more likely to occur if the individual performing the procedure does not practice on a regular basis. If extensive swelling does occur, the procedure must be halted until the swelling has subsided, usually within 24 to 48 hrs. The local and systemic effects of tissue inflammation should be taken into consideration when selection of method of administration is made.

Lastly, analgesia is required for the intratracheal injection technique. The use of certain analgesics may be contraindicated in a particular study. Examples include opioids in behavioural research [6] and NSAIDs in cancer research [7]. However, this should not supersede the use of analgesics [8]. If this is the case, it is recommended that an alternate mode of administration be chosen or a different class of analgesics be utilized.

## Conclusions

In summary, we evaluated three methods of AAV vector administration to the upper and lower respiratory tract in mice: intranasal, intubation, and intratracheal injection. In addition, we presented a modified-intranasal delivery technique (modified-intranasal). All methods of viral vector administration resulted in transduction of the trachea and nasal epithelium, albeit to a lesser extent with the intubation and intratracheal methods of delivery. Intubation was the only method that resulted in transduction of alveoli in the distal most aspect of the lung. The modified-intranasal technique resulted in the most efficient distribution of vector to the upper and lower respiratory tract with the least amount of inter-animal variability and requiring the least amount of training. Technique selection may greatly influence experimental outcomes and should be selected based on a variety of factors including time, skill, and desired target site.

## Methods

### Animals

Six-week-old C57BL/6 male mice were purchased from Charles River Laboratories (St Constant, QC). Mice were housed in groups of four and food (Teklad Global 14% Protein Rodent Maintenance Diet, Indianapolis, USA) and water (tap) were provided ad libitum. Mice were acclimated to the environment for seven days prior to study initiation.

### AAV vector production

Construction of a recombinant AAV vector expressing the reporter gene, human placental alkaline phosphatase (hPLAP), has been described [9]. The packaging plasmid, pDGM6 [10], which encodes the AAV serotype 6 capsid, was kindly provided by Dr. David Russell (University of Washington). AAV vectors and packaging plasmids were propagated in the SURE 2 (Agilent) strain of *Escherichia coli*. AAV vectors were produced by co-transfection of HEK 293 cells with genome and packaging plasmid as described previously [11]. AAV vector titers were determined by qPCR as described [5]. Each animal was given 1 × 10^11^ vg of A_JE_U3CBA-AP, which expresses hPLAP under a composite promoter (Yu *et al.,* manuscript submitted) comprised of the JE enhancer [9] and U3 promoter from Jaagsiekte sheep retrovirus (JSRV) plus the beta-actin promoter, by one of the four delivery methods. Note that the study was performed using A_JE_U3CBA-AP vector from one production batch.

### Intranasal Technique

Mice were anesthetized (induced and maintained) individually inside a feline nose cone with 2.5% isoflurane (Aerrane, Baxter Corp, Mississauga, ON) delivered in O_2_ (1 L/min). Immediately prior to delivering the vector, the mouse was removed from the nose cone and placed in dorsal recumbency (Fig. 1a). A preliminary volume of 40 μl of vector was delivered drop wise over the nares and passively inhaled. The mice were permitted to recover from anesthesia for 10 min in a recovery cage and the procedure repeated (total of 80 μl of vector administered).

### Modified-intranasal Technique

Mice were anesthetized (induced and maintained) with 2.5% isoflurane in O_2_ (1 L/min) as described for the intranasal technique. Immediately prior to delivering the vector, the mouse was removed from the nose cone and gently restrained by the scruff at a 45° angle using the right hand and the mouth gently pinched shut using the thumb and forefinger of the left hand so as to prevent mouth breathing. Once the mouse was restrained and the mouth properly covered, a second individual delivered a preliminary volume of 40 μl of vector drop wise over the nares and the drops passively inhaled (Fig. 1b). The mouth was uncovered once all of the liquid was inhaled. The mouse was maintained in this position for approximately 30 s after vector delivery to allow for passive distribution of vector into the lower respiratory tract. The mice were permitted to recover for approximately 10 min in a recovery cage and the procedure repeated (total of 80 μl of vector administered).

### Intubation Technique

Mice were anesthetized (induced and maintained) with 2.5% isoflurane in O_2_ (1 L/min) and positioned in dorsal recumbency on a metal board oriented at a 45° angle with the rostral end towards the user (Fig. 1c). The upper incisor teeth were hooked under an elastic band attached to the metal board. Mice were exposed to isoflurane throughout the procedure via polyurethane connector tubing placed over the nares. A flexible, high-intensity light source (e.g. Fiber-Lite® high intensity fiber optic flexible illuminator, Dolan-Jenner Industries, MA United States) was positioned 1 cm from the ventral aspect of the sternum. The light shines rostrally through the trachea, allowing for differentiation of the lumen of the trachea from that of the esophagus. A set of serrated blunt nose curved (20° angle) stainless steep forceps (Fine Scientific Tools, item# 11051-10) were used to gently extend the mouse’s tongue out and to the side of the mouth allowing for direct visualization of the epiglottis and larynx. The tongue was held in this position throughout the procedure. Next, blunt stainless steel forceps bent at a 90° angle (Fine Scientific Tools, item# 11052-10) were inserted under the mouse’s tongue and carefully lifted dorsally to allow for greater visualization and access to the trachea. At this point, small adjustments were made to visualize the epiglottis. Using the dominant hand, a 22-gauge catheter was placed between the index and middle finger and stabilized with the thumb in order to direct the catheter through the mouth and larynx into the trachea, stopping just before the bifurcation of the trachea (Fig. 1c). The metal stylette was quickly removed and the polyurethane tubing attached to the catheter opening to allow for continued delivery of isoflurane. Once properly intubated, the polyurethane tube was briefly removed from the end of the catheter, and using a P200 pipette, 80 μl of vector was carefully injected into the catheter as the mouse inhaled. Note that approximately 100 μl of air was drawn up into the pipette tip prior to drawing up 80 μl of vector so as to prevent loss of vector and to promote delivery of the vector deep into the lungs. The mouse was left in this position for 5 min to allow for passive distribution of vector into the lower respiratory tract.

### Intratracheal Injection Technique

Mice were anesthetized (induced and maintained) with 2.5% isoflurane in O_2_ (1 L/min) and positioned in dorsal recumbency on a metal board oriented at a 45° angle with the rostral end away from the user. A 0.5 cm incision was made through the skin, directly cranial to the manubrium. Subcutaneous tissue was carefully dissected away and tissue forceps were used to gently lateralize salivary glands to visualize the trachea (Fig. 1d). Note that applying gentle downward pressure with forceps onto either side of the trachea allows for better visualization of the tracheal rings, which is essential to confirming the appropriate injection location. Using a 29-gauge tuberculin syringe (BD, New Jersey USA) at a 30-45° angle, 80 μl of virus was injected into the trachea. The skin was then closed with 5-0 poliglecaprone 25 (Monocryl, Ethicon, Sommerville, USA) in a single cruciate pattern.

### Tissue Preparation and Staining

Mice were euthanized three weeks following vector administration. The lung, trachea, and nasal cavity were harvested and fixed in 2% paraformaldehyde for 24 to 48 hr at room temperature. After washing three times in phosphate buffered saline (PBS) to remove the fixative, tissues were transferred to 65 °C PBS, incubated for 1 hr in a water bath at 65 °C to inactivate endogenous alkaline phosphatase and then stained for hPLAP expression as previously described [9]. After gross images were obtained, tissues were paraffin embedded and sectioned at 4 μm.

### Genomic DNA extraction and AAV copy number analysis

Five serial sections of 8-micrometer thickness each per sample were taken from each animal using a standard microtome with disposable DNA-RNA free blades. Tissue slices were obtained after trimming to remove tissue exposed to oxygen and to ensure that all five lung lobes were contained within the same plane in the tissue slices. The five tissue slices were placed into a sterile 1.5 ml microfuge tube and genomic DNA was isolated using the QIAamp DNA FFPE Tissue Kit according to the manufacturer’s instructions. DNA was quantified using the Qubit fluorometer and PicoGreen quantification reagents (Invitrogen). Vector genome copy number per ng of genomic DNA was determined by Taqman qPCR starting from 100 ng of mouse gDNA by amplifying the AAV ITRs and mouse beta actin (*Actb*) as a mouse housekeeping gene. The absolute amount of each gene was obtained by referring to a standard curve consisting of a 10-fold serial dilution of a plasmid containing two copies of the AAV ITR and one copy of the *Actb* gene sequence (six concentrations, ranging from 10-10^−6^ copies). Real-time PCR assays were conducted using a StepOnePlus Real-Time PCR system from Thermo Fisher Scientific Inc. Ten μl reactions containing 5 μl 2 × Taqman Master Mix (PerFeCta FastMix II from Quanta BioScience (Cat. No. 95119-250), 1ul of PCR primer/probes (final concentration of primer was 500 nM and probe was 250 nM) and 4 μl of plasmid DNA in serial dilutions. The run conditions were as follows: 30 s at 95 °C polymerase activation step, followed by 35 to 40 cycles of a two-step qPCR (3 s of 95 °C denaturation, 30 s of 60 °C combined annealing/extension). Primers and probes were purchased from IDT and used as follows: AAV ITR, 5′-GGAACCCCTAGTGATGGAGTT-3′ (forward), 5′-CGGCCTCAGTGAGCGA-3′ (reverse), 56-FAM/CACTCCCTC/Zen/T CTGCGCGCTCG/3IABkFQ (probe) [5]; and mouse Actb PrimeTime Std® qPCR Assay from IDT (Mm.PT.39a.22214843.g).

### Statistical analysis

Data were analyzed with Graphpad Prism version 5 (Graphpad Software) and expressed as mean ± standard error of the mean. Statistical differences between mean values were tested using one-way ANOVA followed by the Tukey’s post-hoc test. Differences between values were considered to be significant with: *P < 0.05.

## Additional files

Additional file 1: Figure S1. Histological analysis of alkaline phosphatase staining in the lungs of mice 21 days after delivering 1x10^11^ vg of an AAV6 vector expressing hPLAP by the intranasal method of administration. Tissue sections of all five lung lobes were stained for hPLAP expression and counterstained with nuclear fast red and representative images are shown. Images were taken at 1.5x magnification. Scale bar = 1 mm. (TIF 6183 kb)

Additional file 2: Figure S2. Histological analysis of alkaline phosphatase staining in the lungs of mice 21 days after delivering 1x10^11^ vg of an AAV6 vector expressing hPLAP by the modified intranasal method of administration. Tissue sections of all five lung lobes were stained for hPLAP expression and counterstained with nuclear fast red and representative images are shown. Images were taken at 1.5x magnification. Scale bar = 1 mm. (TIF 8321 kb)

Additional file 3: Figure S3. Histological analysis of alkaline phosphatase staining in the lungs of mice 21 days after delivering 1x10^11^ vg of an AAV6 vector expressing hPLAP by the intubation method of administration. Tissue sections of all five lung lobes were stained for hPLAP expression and counterstained with nuclear fast red and representative images are shown. Images were taken at 1.5x magnification. Scale bar = 1 mm. (TIF 7519 kb)

Additional file 4: Figure S4. Histological analysis of alkaline phosphatase staining in the lungs of mice 21 days after delivering 1x10^11^ vg of an AAV6 vector expressing hPLAP by the intratracheal injection method of administration. Tissue sections of all five lung lobes were stained for hPLAP expression and counterstained with nuclear fast red and representative images are shown. Images were taken at 1.5x magnification. Scale bar = 1 mm. (TIF 7928 kb)

Additional file 5: Figure S5. Histological analysis of alkaline phosphatase staining in the lungs and trachea of mice 21 days after delivering 1x10^11^ vg of an AAV6 vector expressing hPLAP using four different methods of administration. Viral vectors were administered by the intranasal (A and E), modified-intranasal (B and F), intubation (C and G), or intratracheal injection (D and H) method. Tissue sections of one representative lung lobe stained for hPLAP expression and counterstained with nuclear fast red are shown at 20x magnification (A-D; scale bar = 50 μM). Tissue sections of one representative trachea stained for hPLAP expression and counterstained with nuclear fast red are shown at 20x magnification (E-H; scale bar = 50 μM). (TIF 17789 kb)
