# Supplementary figures and images for: AAV vector distribution in the mouse respiratory tract following four different methods of administration
Source: BMC Biotechnol. 2017 May 15;17:43. doi: 10.1186/s12896-017-0365-2 (PMC5433059; doi:10.1186/s12896-017-0365-2)

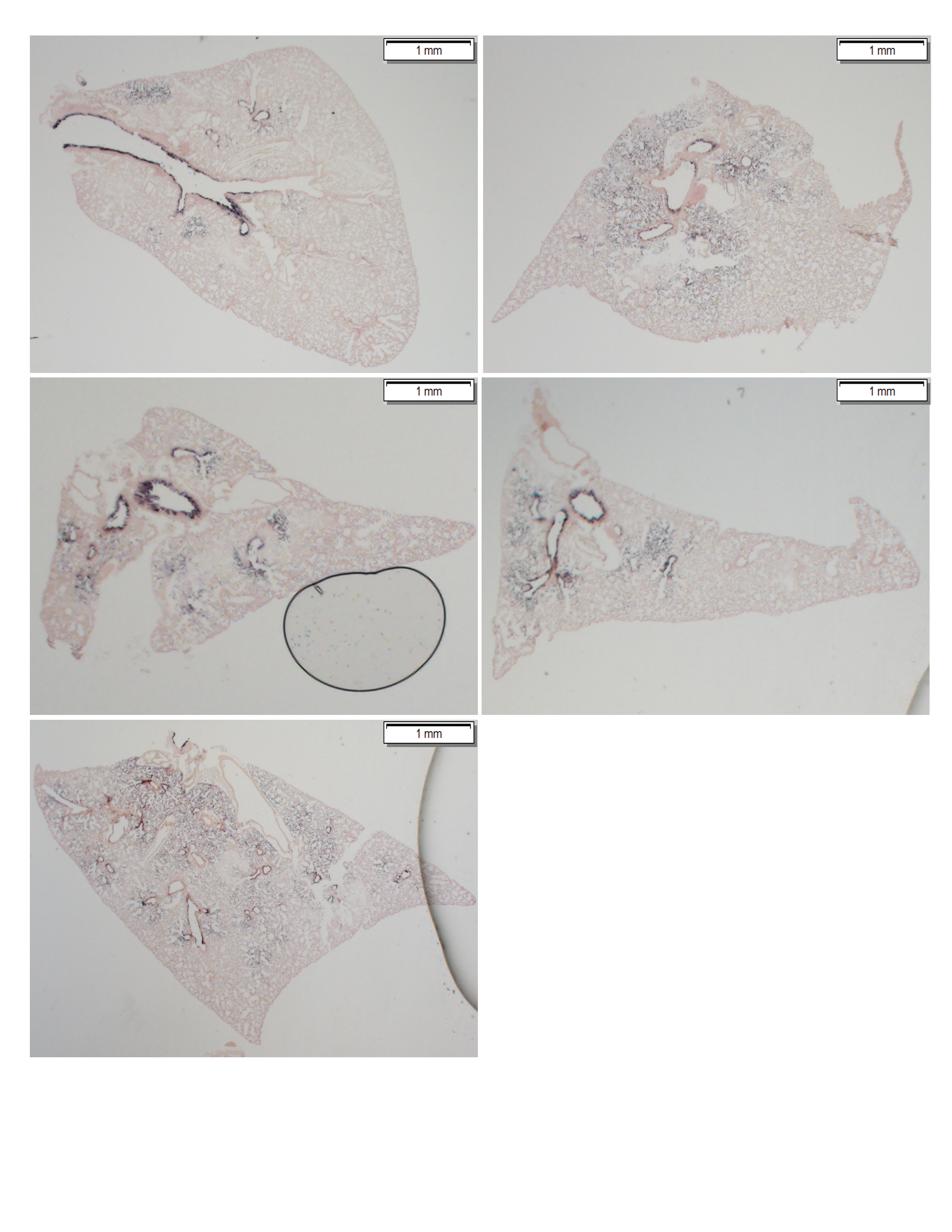

Supplement: Supplementary file 1 — Histological analysis of alkaline phosphatase staining in the lungs of mice 21 days after delivering 1x10^11^ vg of an AAV6 vector expressing hPLAP by the intranasal method of administration. Tissue sections of all five lung lobes were stained for hPLAP expression and counterstained with nuclear fast red and representative images are shown. Images were taken at 1.5x magnification. Scale bar = 1 mm. (TIF 6183 kb) [file 12896_2017_365_MOESM1_ESM.tif]

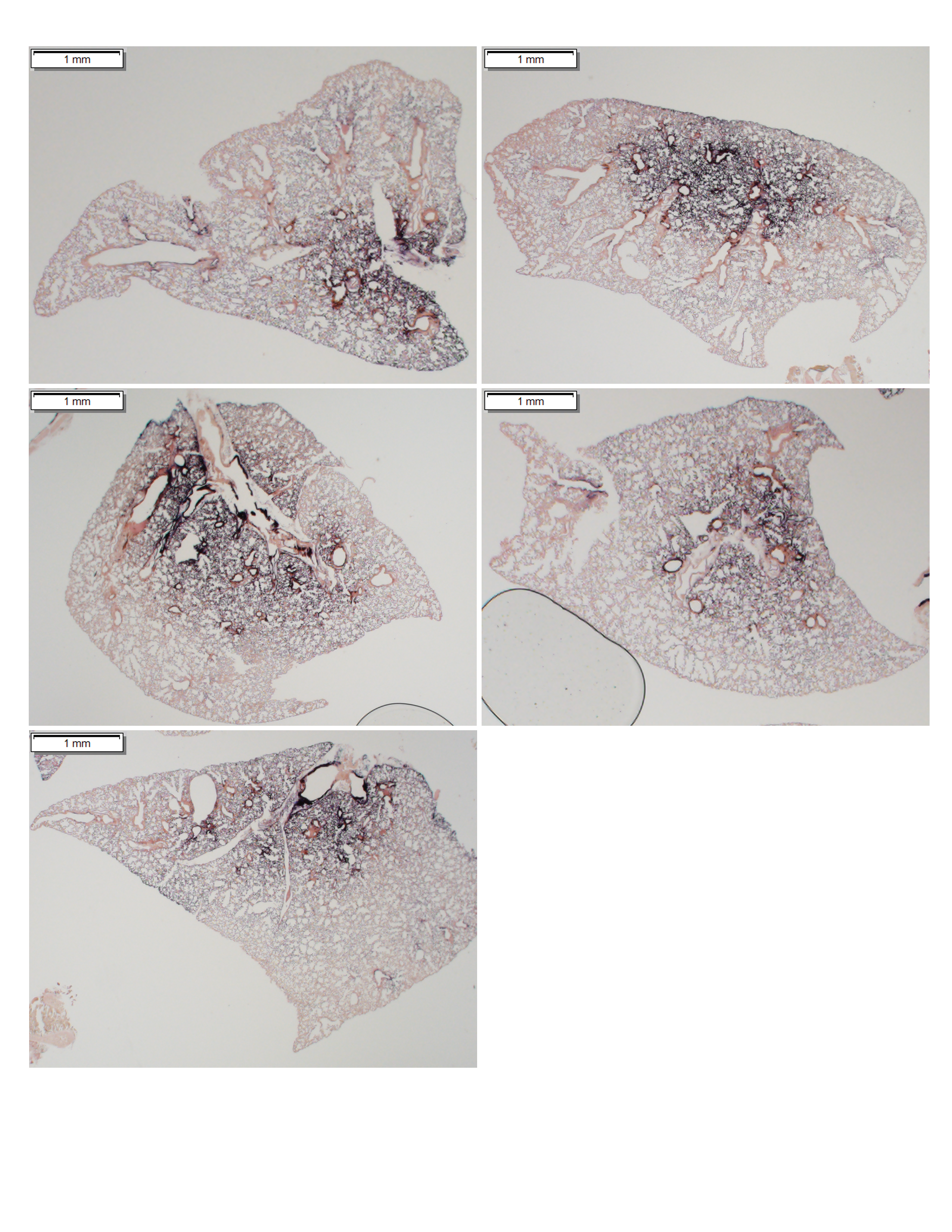

Supplement: Supplementary file 2 — Histological analysis of alkaline phosphatase staining in the lungs of mice 21 days after delivering 1x10^11^ vg of an AAV6 vector expressing hPLAP by the modified intranasal method of administration. Tissue sections of all five lung lobes were stained for hPLAP expression and counterstained with nuclear fast red and representative images are shown. Images were taken at 1.5x magnification. Scale bar = 1 mm. (TIF 8321 kb) [file 12896_2017_365_MOESM2_ESM.tif]

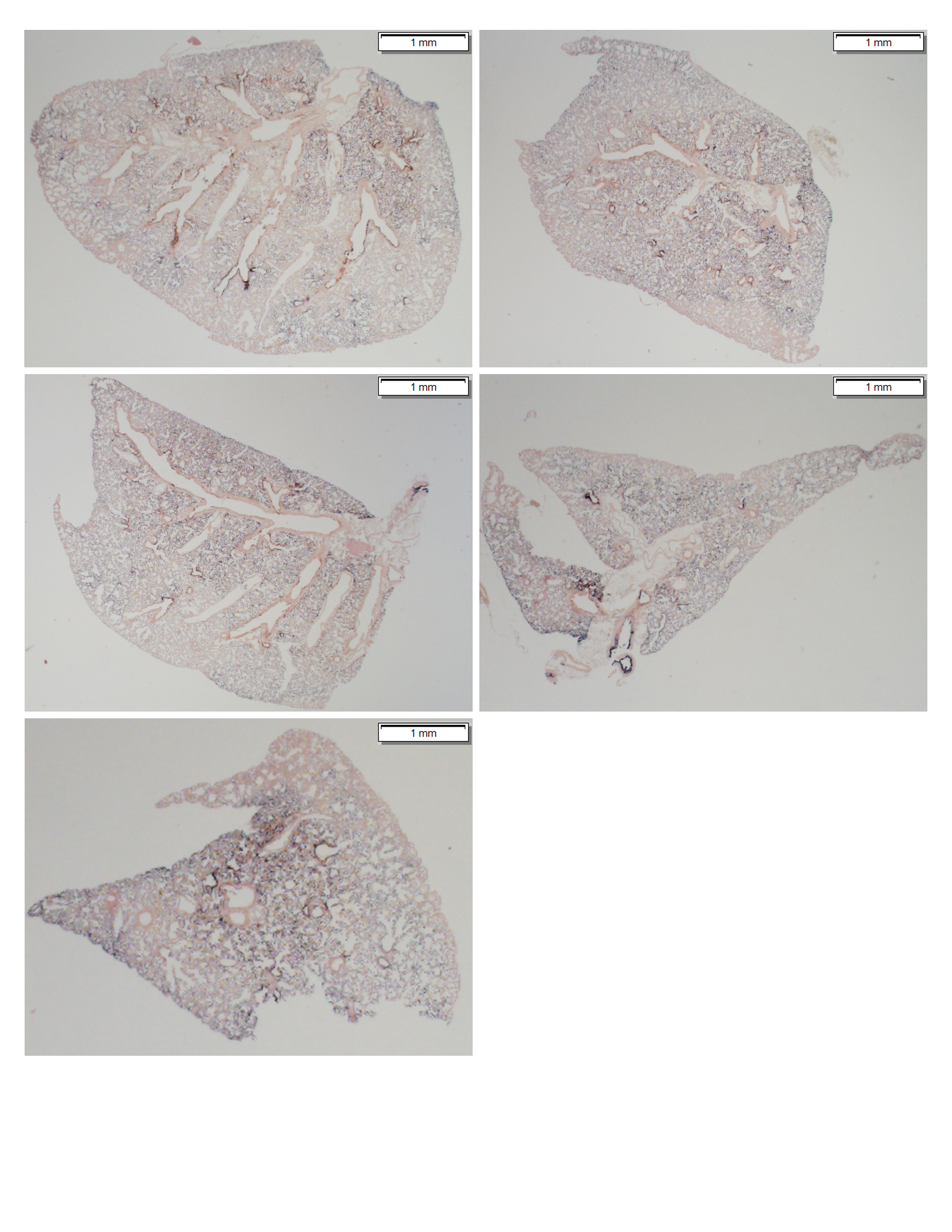

Supplement: Supplementary file 3 — Histological analysis of alkaline phosphatase staining in the lungs of mice 21 days after delivering 1x10^11^ vg of an AAV6 vector expressing hPLAP by the intubation method of administration. Tissue sections of all five lung lobes were stained for hPLAP expression and counterstained with nuclear fast red and representative images are shown. Images were taken at 1.5x magnification. Scale bar = 1 mm. (TIF 7519 kb) [file 12896_2017_365_MOESM3_ESM.tif]

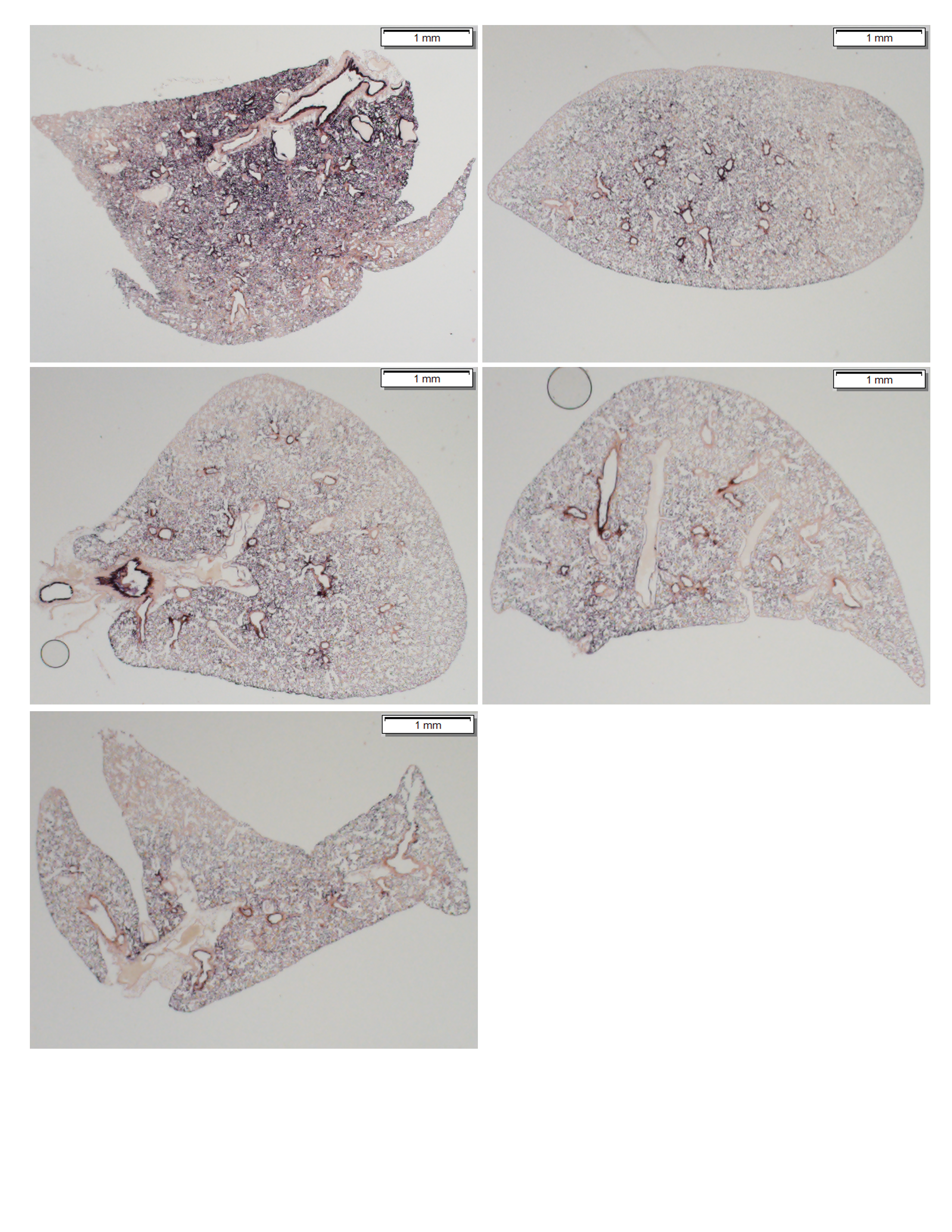

Supplement: Supplementary file 4 — Histological analysis of alkaline phosphatase staining in the lungs of mice 21 days after delivering 1x10^11^ vg of an AAV6 vector expressing hPLAP by the intratracheal injection method of administration. Tissue sections of all five lung lobes were stained for hPLAP expression and counterstained with nuclear fast red and representative images are shown. Images were taken at 1.5x magnification. Scale bar = 1 mm. (TIF 7928 kb) [file 12896_2017_365_MOESM4_ESM.tif]

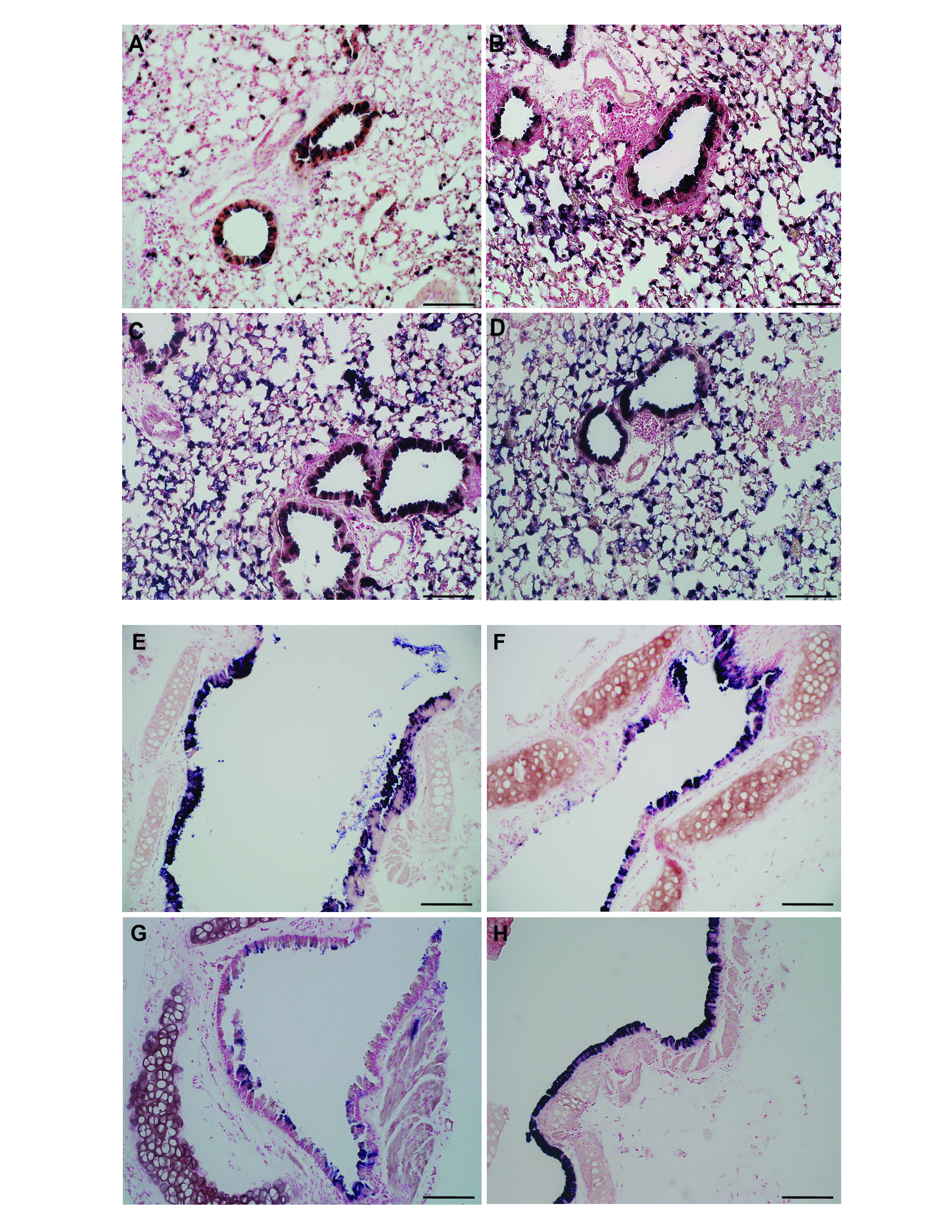

Supplement: Supplementary file 5 — Histological analysis of alkaline phosphatase staining in the lungs and trachea of mice 21 days after delivering 1x10^11^ vg of an AAV6 vector expressing hPLAP using four different methods of administration. Viral vectors were administered by the intranasal (A and E), modified-intranasal (B and F), intubation (C and G), or intratracheal injection (D and H) method. Tissue sections of one representative lung lobe stained for hPLAP expression and counterstained with nuclear fast red are shown at 20x magnification (A-D; scale bar = 50 μM). Tissue sections of one representative trachea stained for hPLAP expression and counterstained with nuclear fast red are shown at 20x magnification (E-H; scale bar = 50 μM). (TIF 17789 kb) [file 12896_2017_365_MOESM5_ESM.tif]
